# Addendum to: A theoretical study of top-mass measurements at the LHC using NLO+PS generators of increasing accuracy

**DOI:** 10.1140/epjc/s10052-019-7336-9

**Published:** 2019-10-19

**Authors:** Silvia Ferrario Ravasio, Tomáš Ježo, Paolo Nason, Carlo Oleari

**Affiliations:** 1 IPPP, Department of Physics, Durham University, Durham, UK; 2 Physics Institute, Universität Zürich, Zurich, Switzerland; 3 Università di Milano-Bicocca and INFN, Sezione di Milano-Bicocca, Piazza della Scienza 3, 20126 Milan, Italy

## Abstract

This paper is a follow-up of Ref. Ferrario Ravasio et al. (Eur Phys J C 78:458, 2018. arXiv:1801.03944), where we studied the impact of next-to-leading order calculations merged with parton shower generators (NLO+PS) of increasing accuracy in the extraction of the top mass at hadron colliders. Here we examined results obtained with the older (fortran-based) shower generators Pythia6.4 and Herwig6.5. Our findings are in line with what we found in Ref. Ferrario Ravasio et al. (2018) with the new, c++-based, generators Pythia8.2 and Herwig7.1.

## Introduction

In Ref. [1] we considered three NLO+PS generators for $t\bar{t}$ production, $hvq$ [2], $t\bar{t}dec$ [3], and $b\bar{b}4\ell$ [4], implemented in the POWHEG BOX [5–8], interfaced with either Pythia8.2 (Py8.2) [9] or Herwig7.1 (Hw7.1) [10, 11]. We focused particularly on an observable that mimics those used in direct top mass measurements, but also included in our study the proposed top mass measurements from the peak energy of the *b* jet [12] and from the class of leptonic observables suggested in Ref. [13]. We found large differences between predictions obtained using the two parton shower programs. In particular, while results obtained with the three NLO+PS generators interfaced to Py8.2 are fairly consistent among each other, large differences are found if they are interfaced to Hw7.1.

In this addendum we discuss the results obtained with the older, fortran-based versions of the Pythia and Herwig codes. Our purpose is to see if the effects that we have seen are specific to the new implementations, or were already present in the old ones. We briefly recall the characteristics of the older generators:

- Pythia6.4 (Py6.4) [14]: implements a $p_{\mathchoice{\displaystyle }{\scriptstyle }{\scriptscriptstyle }{\scriptscriptstyle } \mathrm T}$-ordered shower, making use of the same algorithm adopted in Py8.2. The older and new codes have both an interleaved radiation scheme between the initial-state radiation and the multi-parton interactions (MPI). In Py8.2, final-state radiation is also interleaved, and different models of colour reconnection are also offered.
- Herwig6.5 [15] with Jimmy 4.31 [16] (Hw6.5): implements an angular-ordered shower. However, the showering variables are different from those adopted in Hw7.1. In the latter code, a boost-invariant set of showering variables was introduced, as described in Ref. [17]. Thus the older and newer schemes are fully equivalent only in the strict collinear limits. The two versions of Herwig implement the PS and the perturbative part of the MPI in a similar manner. The non-perturbative part of the MPI, instead, has been completely redesigned [18]. Similarly to Pythia, colour-reconnection effects are properly included only in the recent versions of Herwig [19].

By including Herwig6.5 and Pythia6.4 we exhaust all possible shower generators that can be interfaced to our NLO ones, since these are the only ones that implement the Les Houches Interface for User Processes [20].

The purpose of Ref. [1] was to understand and estimate uncertainties in top-mass measurements by comparing generators of different formal accuracy, i.e. the $b\bar{b}4\ell$, $t\bar{t}dec$ and $hvq$ ones. In doing so, it was found that switching the shower programs (to which the three NLO generators are interfaced to) yields large differences in the results, in spite of the fact that the different shower programs have fairly similar formal accuracy. These differences must be ascribed to the fact that different shower Monte Carlo programs may differ widely in their modeling of subleading effects, like the non-collinear radiation, the colour-reconnection schemes and the models for hadronization and multi-parton interactions. It is thus natural to extend the study of Ref. [1] with the inclusion of other shower generators, in order to further explore the impact of these differences.

We are aware of the fact that the c++ and fortran versions of the generators we are considering undoubtedly share some similarities, since the latter are the ancestors of the former ones. In spite of this, we found non-negligible differences, that we will discuss in the following.

In our previous work, we have seen that the two generators $b\bar{b}4\ell$ and $t\bar{t}dec$ yield fairly consistent results for the mass of the reconstructed top and the *b*-jet energy. In the case of leptonic observables, the differences between $b\bar{b}4\ell$ and $t\bar{t}dec$ within the same shower model are generally much smaller than the differences between the different shower models for the same NLO generator. The largest difference between $b\bar{b}4\ell$ and $t\bar{t}dec$ appears in association with Herwig7.1, and is around 1.5 GeV, while the difference between Herwig7.1 and Pythia8.2 in $t\bar{t}dec$ is about 2.5 GeV (see Fig. 17 of Ref. [1]). For these reasons, we only consider the $hvq$ and $b\bar{b}4\ell$ generators in this addendum.

## Interface to POWHEG BOX

In this section we briefly describe the matching of $b\bar{b}4\ell$ and $hvq$ to both Py6.4 and Hw6.5. The matching to Py8.2 and Hw7.1 is detailed in Ref. [1].

### Pythia6.4

Py6.4 implements both a $p_{\mathchoice{\displaystyle }{\scriptstyle }{\scriptscriptstyle }{\scriptscriptstyle } \mathrm T}$ and a virtuality-ordered PS. Here, we employ the $p_{\mathchoice{\displaystyle }{\scriptstyle }{\scriptscriptstyle }{\scriptscriptstyle } \mathrm T}$-ordered shower with the Perugia tune (PYTUNE(320)) [21].

We setup Py6.4 in such a way that the $p_{\mathchoice{\displaystyle }{\scriptstyle }{\scriptscriptstyle }{\scriptscriptstyle } \mathrm T}$ of radiation in the shower is limited by the scalup parameter of the Les Houches Interface for User Processes [20], as is usually done in POWHEG. This is at variance with the Perugia tune settings, that requires $p_{\mathchoice{\displaystyle }{\scriptstyle }{\scriptscriptstyle }{\scriptscriptstyle } \mathrm T}$ to be smaller than scalup divided by $\sqrt{2}$.^1^

The matching of shower emissions in the production process relies on the default behaviour of POWHEG, i.e. the shower evolution starts at scalup. In the decays, a different scale must be adopted, and thus it requires a custom veto prescription in $b\bar{b}4\ell$. We implement it using two methods, both analogous to what we did in order to match Py8.2 to $b\bar{b}4\ell$ in Ref. [1]:

1. Each time Pythia6.4 generates an emission off the top (or anti-top), we compute its transverse momentum according to the POWHEG definition. If it is larger than the transverse momentum of the emission generated by the POWHEG BOX, we abandon the current shower, and restart a shower from the same Les Houches event. This represents our default method. We label it as the “FSR” veto, in full analogy with the notation adopted for Py8.2.
2. Since we employ a $p_{\mathchoice{\displaystyle }{\scriptstyle }{\scriptscriptstyle }{\scriptscriptstyle } \mathrm T}$-ordered shower, we can also simply require the shower to start at a given transverse momentum, that we set equal to the transverse momentum of the corresponding POWHEG emission. This veto procedure will be referred to as the “SR” method, as we did with the analogous method that we adopted in Py8.2.

### Herwig6.5

For Hw6+Jimmy we adopted the ATLAS AUET2 tune [22]. The Herwig shower is ordered in angle and not in $p_{\mathchoice{\displaystyle }{\scriptstyle }{\scriptscriptstyle }{\scriptscriptstyle } \mathrm T}$. Therefore all the emissions with transverse momentum larger than that of the POWHEG emission must be vetoed. Both Herwig versions already enforce this veto for the production part of the process. Similarly to Py6.4, extra care is required for emissions from the top-decay products, when interfaced with $b\bar{b}4\ell$.

In our previous work, two procedures were devised to veto extra Hw7.1 emissions. Both of them use the $p_{\mathchoice{\displaystyle }{\scriptstyle }{\scriptscriptstyle }{\scriptscriptstyle } \mathrm T}$ of the POWHEG emission as an upper bound, either on the $p_{\mathchoice{\displaystyle }{\scriptstyle }{\scriptscriptstyle }{\scriptscriptstyle } \mathrm T}$ of each branching at the end of the showering phase (FullShowerVeto), or on the shower evolution scale during the showering phase (ShowerVeto). Unfortunately, the Hw6.5 event record (as for Py6.4) does not contain information regarding the branching of the partons, i.e. it is not possible to reconstruct the emission’s history after the shower is completed, in contrast to the new version of the code. Therefore, we only implemented the analogue of the Hw7.1 ShowerVeto method which proceeds as follows: when an emission off a top resonance is generated, if its $p_{\mathchoice{\displaystyle }{\scriptstyle }{\scriptscriptstyle }{\scriptscriptstyle } \mathrm T}$ (defined in terms of Herwig variables) is larger than that of the POWHEG emission, the branching is discarded and the evolution continues from the scale of this discarded emission.

## Hadronic observables: NLO+PS results

In this section we compare predictions for hadronic observables at the NLO+PS level, i.e. without the inclusion of MPI and of hadronization effects. Our aim is to assess differences of perturbative origin and, in particular, due to the NLO+PS matching.

### Pythia6.4 versus Pythia8.2

We begin by comparing the predictions obtained with Py6.4 and Py8.2, which both implement a dipole-like algorithm for final-state showers.

**Fig. 1:**
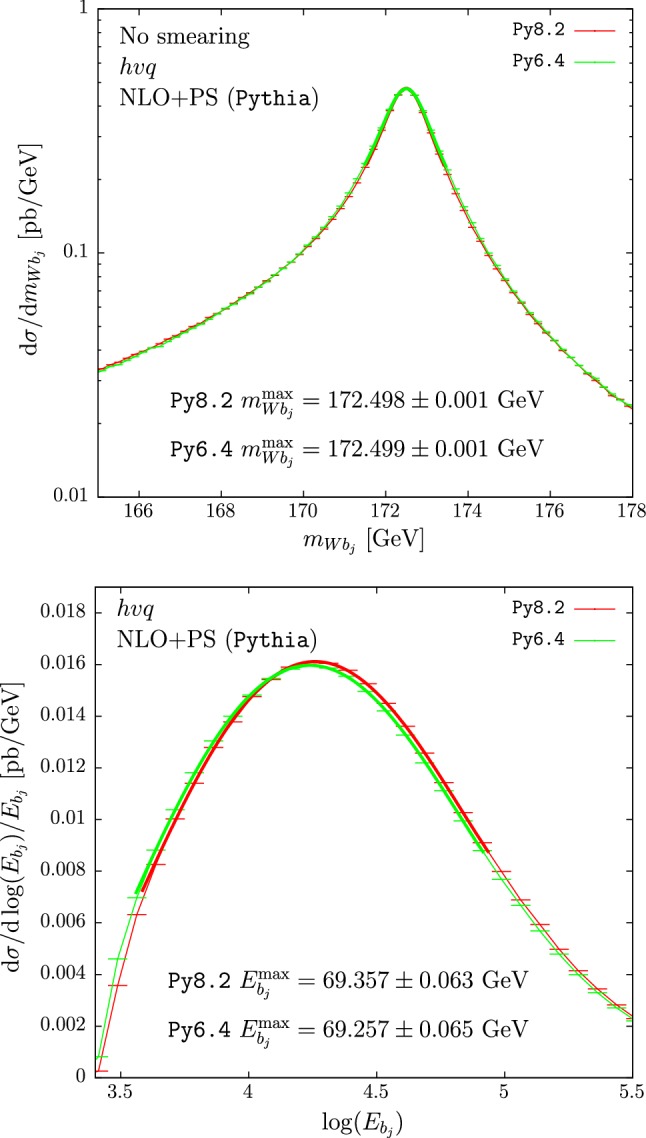
Reconstructed-top mass (upper pane) and $b$-jet energy distribution (lower pane) obtained with the $hvq$ generator interfaced to Py8.2 (red) and to Py6.4 (green). Hadronization and MPI effects are not included

In Ref. [1] we made use of a smearing procedure to simulate experimental resolution effects. We begin by examining results obtained without applying any smearing.

The distributions of the reconstructed-top mass and of the $b$-jet energy using $hvq$ matched to the two versions of Pythia are shown in the upper and lower panes of Fig. 1, respectively. The two curves for the reconstructed-top mass are almost indistinguishable. Also the peak positions of the $b$-jet energy spectra agree remarkably well, despite some small differences in shape, leading to a displacement of the extracted top-mass for this observable of $\approx 200$ MeV.

**Fig. 2:**
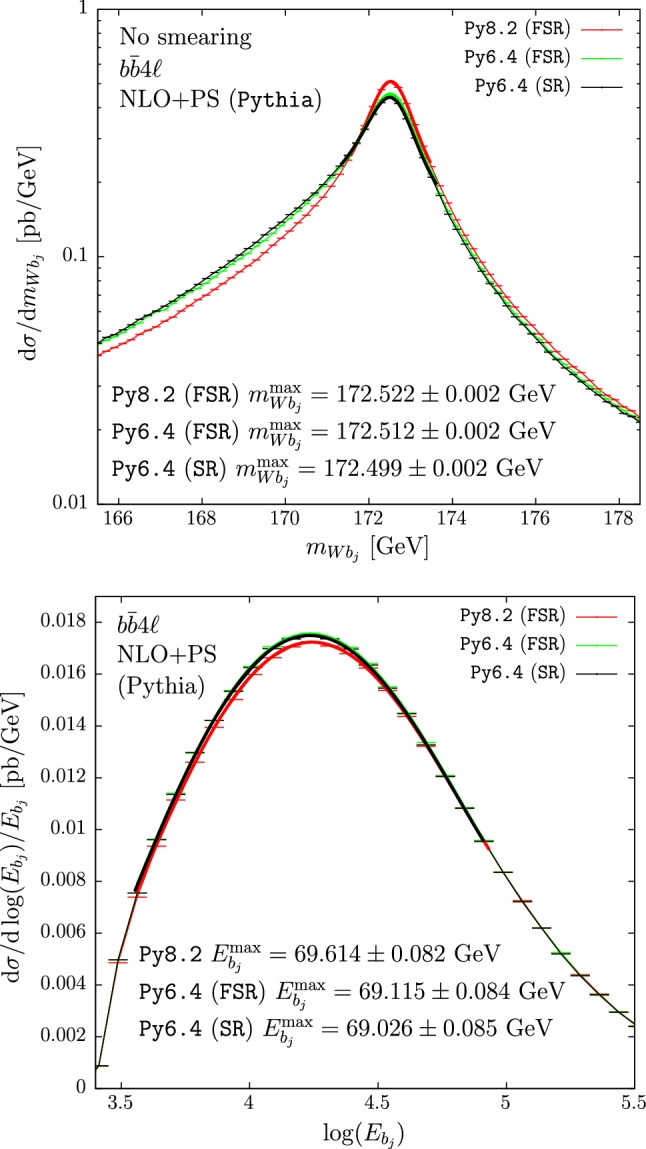
Reconstructed-top mass (upper pane) and $b$-jet energy distributions (lower pane) obtained with the $b\bar{b}4\ell$ generator showered by Py8.2 with the FSR veto scheme (red), and by Py6.4. The two curves for the Py6.4 results are obtained using the FSR veto scheme (green) and the SR veto scheme (black). Hadronization and MPI effects are not included

**Table 1:** Comparisons between the Py8.2 and the Py6.4 results for $m_{Wb_j}^{\max }$, computed with $b\bar{b}4\ell$ and $hvq$, without hadronization or MPI effects, for different values of the jet radius *R*

	$R=0.4$		$R=0.5$		$R=0.6$	
	No smearing	15 GeV smearing	No smearing	15 GeV smearing	No smearing	15 GeV smearing
$b\bar{b}4\ell$+Py8.2 (FSR) (GeV)	$172.509\pm 0.002$	$170.569\pm 0.002$	$172.522\pm 0.002$	$171.403\pm 0.002$	$172.538\pm 0.002$	$172.117\pm 0.002$
$b\bar{b}4\ell$+Py6.4 (FSR) ${}-$ $b\bar{b}4\ell$+Py8.2 (FSR)	$-22 \pm 3$ MeV	$-296 \pm 2$ MeV	$-11 \pm 3$ MeV	$-286 \pm 2$ MeV	$0 \pm 3$ MeV	$-258 \pm 2$ MeV
$b\bar{b}4\ell$+Py6.4 (SR) ${}-$ $b\bar{b}4\ell$+Py8.2 (FSR)	$-36 \pm 3$ MeV	$-360 \pm 2$ MeV	$-23 \pm 3$ MeV	$-342 \pm 2$ MeV	$-8 \pm 3$ MeV	$-307 \pm 2$ MeV
$hvq$+Py8.2 (GeV)	$172.485\pm 0.001$	$170.518\pm 0.001$	$172.498\pm 0.001$	$171.315\pm 0.001$	$172.513\pm 0.001$	$171.996\pm 0.001$
$hvq$+Py6.4 ${}-$ $hvq$+Py8.2	$-11 \pm 2$ MeV	$+ 76 \pm 2$ MeV	$+ 1 \pm 2$ MeV	$+ 69 \pm 2$ MeV	$+ 13 \pm 2$ MeV	$+ 69 \pm 2$ MeV

In Fig. 2 we plot the distributions obtained using the $b\bar{b}4\ell$ generator. The results for the $m_{Wb_j}$ spectrum obtained with Py6.4 show an enhancement in the low-mass region with respect to the Py8.2 distribution, irrespective of the veto scheme used (upper pane). Nevertheless there is no appreciable shift in the peak-position.

The shape of the $b$-jet energy spectrum in the proximity of the peak region is instead different for Py8.2 compared to the two results obtained by using Py6.4, with a shift in the maximum of the $b$-jet energy of approximately +0.5 GeV of the former with respect to the latter two results. This shift induces a displacement in the extracted top-mass ($m_t$) of $\approx 1$ GeV.^2^

**Table 2:** Comparisons between the Py8.2 and the Py6.4 results for $E_{b_j}^{\max }$, computed with $b\bar{b}4\ell$ and $hvq$, without hadronization or MPI effects, for different values of the jet radius *R*

	$R=0.4$	$R=0.5$	$R=0.6$
$b\bar{b}4\ell$+Py8.2 (FSR) (GeV)	$67.145\pm 0.086$	$69.614\pm 0.082$	$71.747\pm 0.080$
$b\bar{b}4\ell$+Py6.4 (FSR) ${}-$ $b\bar{b}4\ell$+Py8.2 (FSR)	$-422 \pm 124$ MeV	$-499 \pm 118$ MeV	$-512 \pm 115$ MeV
$b\bar{b}4\ell$+Py6.4 (SR) ${}-$ $b\bar{b}4\ell$+Py8.2 (FSR)	$-455 \pm 123$ MeV	$-588 \pm 118$ MeV	$-543 \pm 114$ MeV
$hvq$+Py8.2 (GeV)	$66.791\pm 0.068$	$69.357\pm 0.063$	$71.598\pm 0.061$
$hvq$+Py6.4 ${}-$ $hvq$+Py8.2	$-24 \pm 95$ MeV	$-100 \pm 91$ MeV	$-133 \pm 87$ MeV

In Tables 1 and 2 we summarize the $m_{Wb_j}$ and $E_{b_j}$ peak positions respectively, obtained for different values of the jet radius varied between 0.4 and 0.6. Table 1 also shows the $m_{Wb_j}$ distribution peak positions when the smearing is applied. An excellent agreement is found between $hvq$+Py6.4 and $hvq$+Py8.2 for $m_{Wb_j}^{\max }$, even after the smearing is applied, and the $E_{b_j}^{\max }$ differences are small, nearly consistent with zero within their statistical errors for all values of *R*.

The low-mass enhancement in the $m_{Wb_j}$ spectrum of the $b\bar{b}4\ell$+Py6.4 generator, with respect to the $b\bar{b}4\ell$+Py8.2 generator, leads to quite large displacements of the peak position once smearing is applied. For our default FSR-veto procedure, the differences between Py8.2 and Py6.4 are roughly 250–300 MeV. The differences of $E_{b_j}^{\max }$ for the two showers used with $b\bar{b}4\ell$ are even larger, of the order of 0.5 GeV for all values of the jet radius. It is interesting to notice that $b\bar{b}4\ell$+Py6.4 and $b\bar{b}4\ell$+Py8.2 can yield such large differences, in spite of the fact that they should implement the same shower model, and now we are not considering hadronization and MPI effects.

The differences in $m_{Wb_j}^{\max }$ and $E_{b_j}^{\max }$ between the $b\bar{b}4\ell$ and $hvq$ generators for $R=0.5$ are reported in Table 3.

We notice that the level of agreement of $m_{Wb_j}^{\max }$ predictions obtained using $b\bar{b}4\ell$ and $hvq$ gets worse in Py6.4 as compared to Py8.2, while the opposite is true for $E_{b_j}^{\max }$.

### Herwig6.5 versus Herwig7.1

We now compare the predictions obtained by showering the NLO+PS results with Hw6.5 and Hw7.1.

In the upper panes of Figs. 3 and 4 we plot the results for $m_{Wb_j}$ obtained with $hvq$ and $b\bar{b}4\ell$. The cross section under the peak is mildly suppressed in Hw6.5 with respect to Hw7.1. This is then compensated by enhancements in the low- and, to a smaller extent, high-tail regions. A small bump is also present at roughly 1 GeV below the peak position when using the $b\bar{b}4\ell$ generator with Hw7.1, also present to a smaller extent when using Hw6.5 instead.^3^ These differences, present already at the shower level, could be ascribed to the fact that the two versions of Herwig adopt slightly different ordering variables.^4^ Despite the presence of these differences, the peak position (at the unsmeared level) in Hw6.5 or Hw7.1, in both $hvq$ and $b\bar{b}4\ell$, is not changed.

In the lower panes of Figs. 3 and 4 we show the results for the $b$-jet energy spectrum. The peak position, when $hvq$ is used, is 250 MeV bigger when showering with Hw6.5 than with Hw7.1, while in the case of $b\bar{b}4\ell$ it has the same magnitude but opposite sign. This affects the extracted top mass by 0.5 GeV.

In Tables 4 and 5 we quote the differences between the two Herwig showers for several values of the jet radii.

We notice that the differences between the c++ and fortran versions of Herwig for $m_{Wb_j}^{\max }$ and $E_{b_j}^{\max }$ are considerably smaller than in the Pythia case, in spite of the fact that the two implementations of the angular-ordered shower in Herwig are completely different.

Conversely to the Pythia case, in Herwig the differences between $b\bar{b}4\ell$ and $hvq$ are quite large, as shown in Table 6.

**Table 3:** Differences between the $b\bar{b}4\ell$ and $hvq$ predictions for $m_{Wb_j}^{\max }$ (with and without smearing) and $E_{b_j}^{\max }$, showered by Py8.2 and Py6.4

	$$b\bar{b}4\ell $$$${}-$$ $$hvq$$, $$R=0.5$$ [MeV]		
	$m_{Wb_j}^{\max }$	$m_{Wb_j}^{\max }$ (smear)	$E_{b_j}^{\max }$
Py8.2 (FSR)	$24 \pm 2$	$89 \pm 2$	$257 \pm 53$
Py6.4 (FSR)	$12 \pm 2$	$-265 \pm 2$	$-147 \pm 106$

**Fig. 3:**
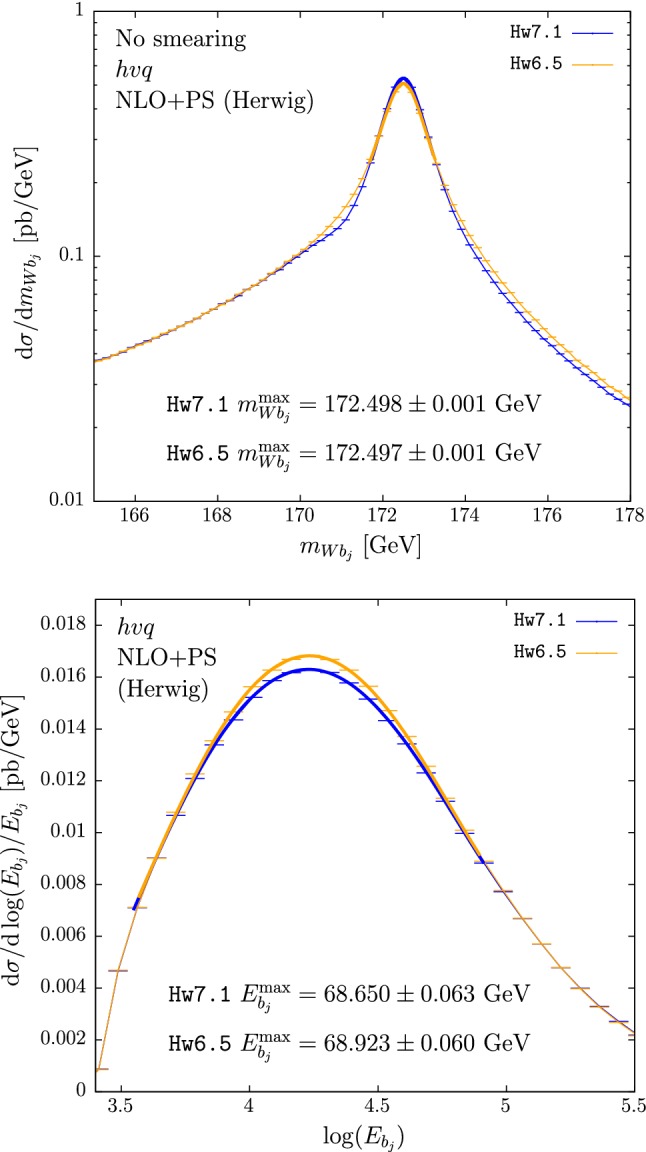
Reconstructed-top mass (upper pane) and *b* jet energy distribution (lower pane) computed with the $hvq$ generator matched to Hw7.1 (blue) and to Hw6.5 (orange). Hadronization and MPI effects are not included

**Fig. 4:**
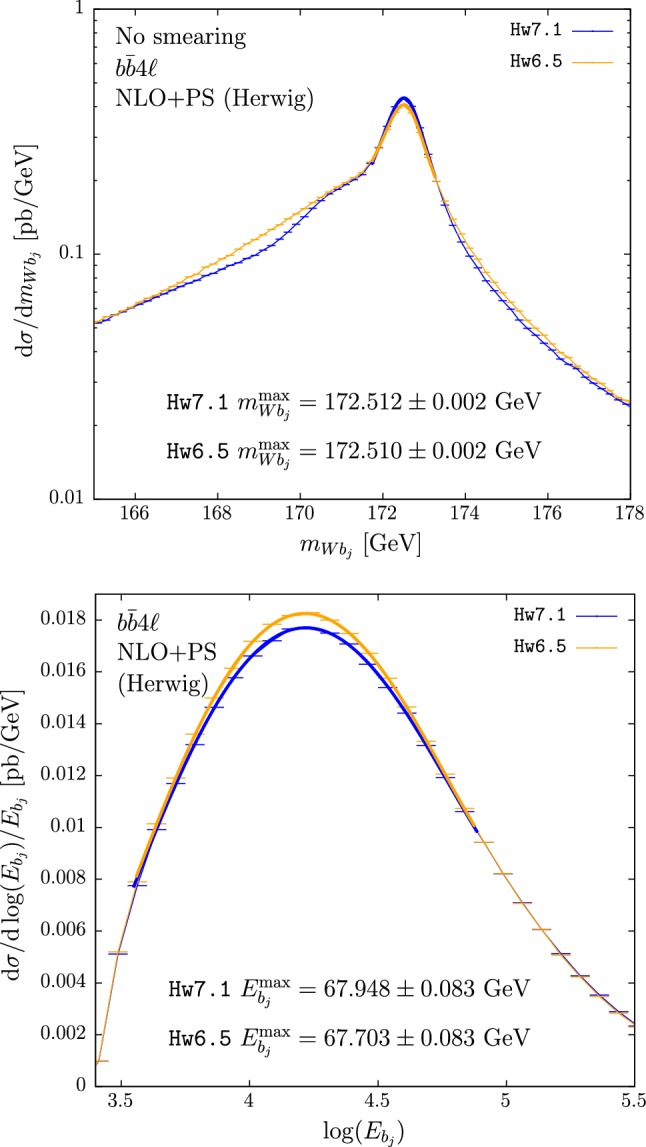
Reconstructed-top mass (upper pane) and *b* jet energy distribution (lower pane) computed with the $b\bar{b}4\ell$ generator matched to Hw7.1 (blue) and to Hw6.5 (orange). Hadronization and MPI effects are not included

**Table 4:** Comparisons between the Hw7.1 and the Hw6.5 results for $m_{Wb_j}^{\max }$, computed with $b\bar{b}4\ell$ and $hvq$, without hadronization or MPI effects, for different values of the jet radius *R*

	$R=0.4$		$R=0.5$		$R=0.6$	
	No smearing	15 GeV smearing	No smearing	15 GeV smearing	No smearing	15 GeV smearing
$b\bar{b}4\ell$+Hw7.1 (GeV)	$172.509\pm 0.002$	$169.699\pm 0.002$	$172.512\pm 0.002$	$170.419\pm 0.002$	$172.517\pm 0.002$	$171.108\pm 0.002$
$b\bar{b}4\ell$+Hw6.5 ${}-$ $b\bar{b}4\ell$+Hw7.1	$-6 \pm 3$ MeV	$-66 \pm 2$ MeV	$-2 \pm 3$ MeV	$+ 34 \pm 2$ MeV	$-4 \pm 3$ MeV	$+ 116 \pm 2$ MeV
$hvq$+Hw7.1 (GeV)	$172.497\pm 0.001$	$170.464\pm 0.001$	$172.498\pm 0.001$	$171.202\pm 0.001$	$172.499\pm 0.001$	$171.867\pm 0.001$
$hvq$+Hw6.5 ${}-$ $hvq$+Hw7.1	$-1 \pm 2$ MeV	$+ 96 \pm 2$ MeV	$-1 \pm 2$ MeV	$+ 81 \pm 2$ MeV	$+ 1 \pm 2$ MeV	$+ 87 \pm 2$ MeV

### Pythia versus Herwig

In Figs. 5 and 6 we plot the variation of $m_{Wb_j}^{\max }$ and $E_{b_j}^{\max }$ (relative to our reference generator combination, i.e. $b\bar{b}4\ell$+Py8.2) obtained with $b\bar{b}4\ell$ and $hvq$, showered by Py8.2, Hw7.1 Py6.4 and Hw6.5.

The shifts for $m_{Wb_j}^{\max }$, without any smearing, are small and comparable when using Hw7.1 or Hw6.5. These are not reported in the figures, and can be obtained from the tables in the appendix.

When the smearing is applied, Hw7.1 and Hw6.5 with $b\bar{b}4\ell$ give comparable negative shifts, around 1 GeV. Instead, with $hvq$, the displacement of the peak position (with respect to the reference values) are around $-100 \div -200$ MeV for Hw7.1, and $0\div -150$ MeV for Hw6.5, for the different jet radii *R*. Since no significant difference between the two Herwig versions was observed in the $b\bar{b}4\ell$ case (where POWHEG generates the hardest emission both in production and decay), and since $hvq$ does not handle radiation in decay, this behaviour is likely to be due to a different treatment of radiation in decay in the two Herwig versions with respect to Pythia.

**Table 5:** Comparisons between the Hw7.1 and the Hw6.5 results for $E_{b_j}^{\max }$, computed with $b\bar{b}4\ell$ and $hvq$, without hadronization or MPI effects, for different values of the jet radius *R*

	$R=0.4$	$R=0.5$	$R=0.6$
$b\bar{b}4\ell$+Hw7.1 (GeV)	$65.847\pm 0.084$	$67.948\pm 0.083$	$69.945\pm 0.082$
$b\bar{b}4\ell$+Hw6.5 ${}-$ $b\bar{b}4\ell$+Hw7.1	$-231 \pm 119$ MeV	$-245 \pm 117$ MeV	$-17 \pm 116$ MeV
$hvq$+Hw7.1 (GeV)	$66.276\pm 0.065$	$68.650\pm 0.063$	$70.819\pm 0.061$
$hvq$+Hw6.5 ${}-$ $hvq$+Hw7.1	$+ 422 \pm 89$ MeV	$+ 273 \pm 87$ MeV	$+ 181 \pm 84$ MeV

As for $E_{b_j}^{\max }$ predictions in Fig. 6, we find minor differences between Hw6.5 and Hw7.1 for $R \ge 0.5$, that go in the direction to amplify the difference with respect to our reference generator. Similarly to $m_{Wb_j}^{\max }$, also in this case the discrepancies between $b\bar{b}4\ell$ and $hvq$ interfaced to the same shower generator are larger for Herwig than for Pythia, both for the older and newer versions.

We interpret the relative consistency of the Hw7.1 and Hw6.5 predictions with the $b\bar{b}4\ell$ generator as a validation of our veto procedures and of the results presented in Ref. [1].

## Hadronic observables: full results

We now summarize the results obtained by showering $hvq$ and $b\bar{b}4\ell$ with the four PS programs at the full level, that is with the MPI and hadronization switched on. The $b\bar{b}4\ell$+Py6.4 results shown here and in the following sections are obtained using the FSR veto.

**Table 6:** Differences between the $b\bar{b}4\ell$ and $hvq$ predictions for $m_{Wb_j}^{\max }$ (with and without smearing) and $E_{b_j}^{\max }$, showered by Hw7.1 and Hw6.5

	$$b\bar{b}4\ell $$$${}-$$ $$hvq$$, $$R=0.5$$ [MeV]		
	$m_{Wb_j}^{\max }$	$m_{Wb_j}^{\max }$ (smear)	$E_{b_j}^{\max }$
Hw7.1	$14 \pm 2$	$-783 \pm 2 2$	$-702 \pm 104$
Hw6.5	$13 \pm 2$	$-829 \pm 2$	$-1220 \pm 102$

**Fig. 5:**
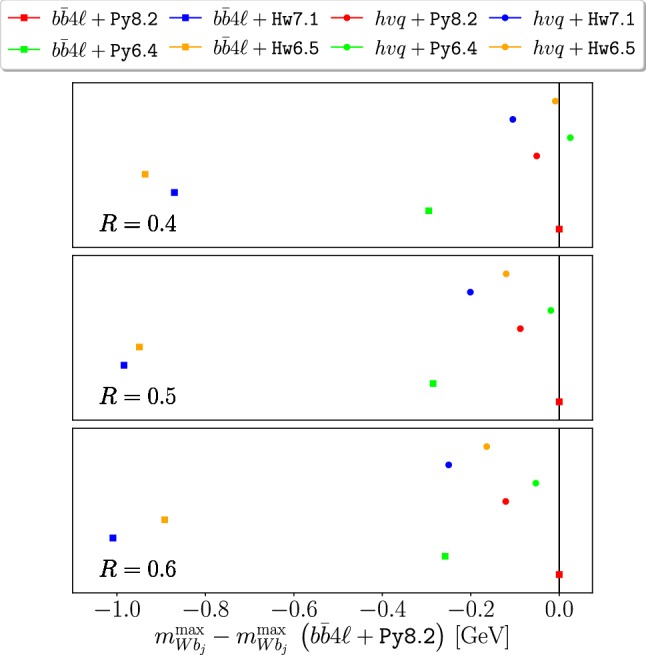
Results for the difference of the $m_{Wb_j}^{\max }$, including a 15 GeV smearing, with respect to our reference generator (i.e. $b\bar{b}4\ell$+Py8.2), at the NLO+PS level using $hvq$ or $b\bar{b}4\ell$, showered by Pythia and Herwig, for different values of jet radius *R*. Hadronization and MPI effects are not included. The numerical values are reported in Table 8. The square/round dots refer to $b\bar{b}4\ell$/$hvq$ results, while the colours correspond to given shower generators

**Fig. 6:**
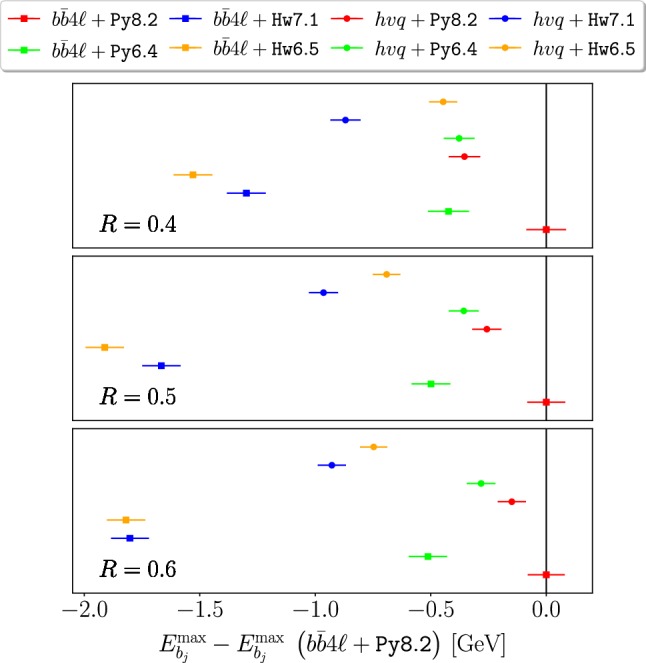
Same as Fig. 5 but for $E_{b_j}^{\max }$

**Fig. 7:**
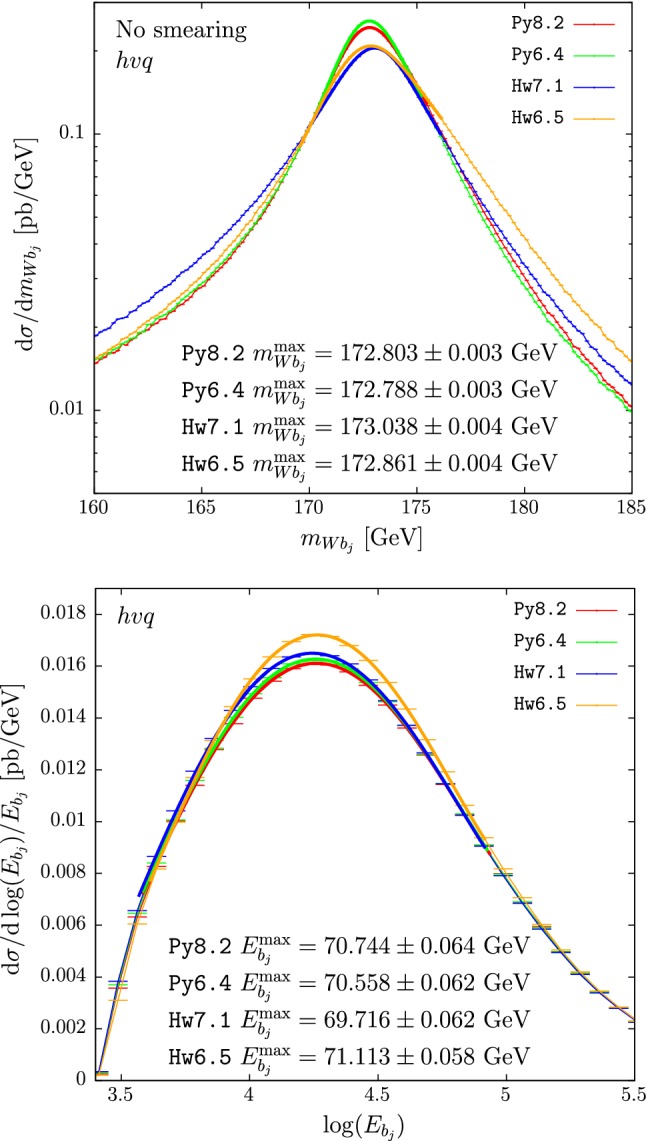
Reconstructed-top mass (upper pane) and *b* jet energy distribution (lower pane) obtained for the $hvq$ generator matched to Py8.2 (red), to Py6.4 (green), Hw7.1 (blue) and Hw6.5 (orange). The hadronization and the underlying event are included

For the $hvq$ generator (see Fig. 7) we find that Py6.4 and Py8.2 yield very similar results. However, we find an appreciable disagreement between Hw7.1 and Hw6.5. We attribute it to different implementations of MPI in the two versions of Herwig, since the predictions agreed rather well at the NLO+PS level for $R\ge 0.5$.^5^

**Fig. 8:**
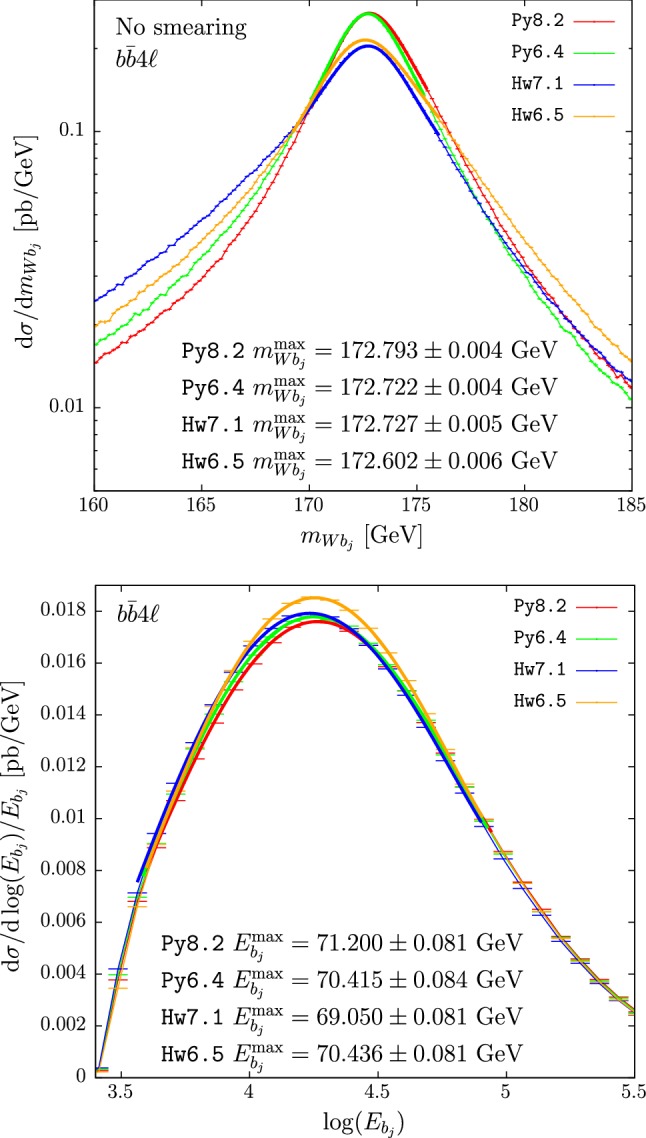
Reconstructed-top mass (upper pane) and *b* jet energy distribution (lower pane) obtained for the $b\bar{b}4\ell$ generator matched to Py8.2 (red), to Pythia6.4 (green), Hw7.1 (blue) and Hw6.5 (orange). The hadronization and the underlying event are included

**Fig. 9:**
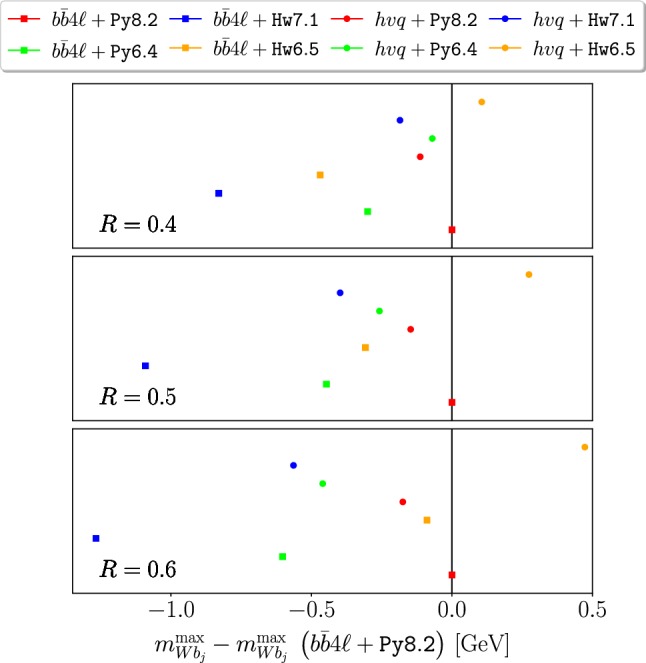
Results for the differences of $m_{Wb_j}^{\max }$, including a 15 GeV smearing, relative to our reference generator, at the full level (i.e. with the inclusion of the MPI and of the hadronization) for different values of jet radius *R*. The numerical values are reported in Table 9. The square/round dots refer to $b\bar{b}4\ell$/$hvq$ results, while the colours correspond to given shower generators

**Fig. 10:**
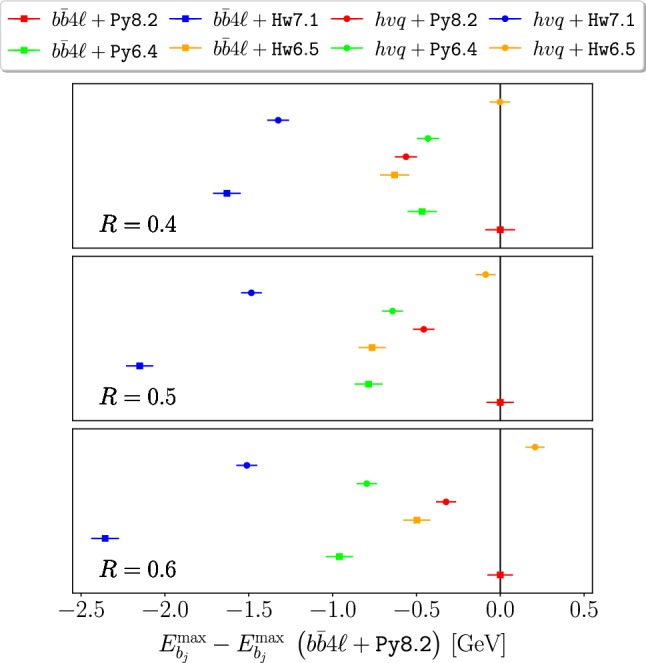
Same as Fig. 9 but for $E_{b_j}^{\max }$

If the $b\bar{b}4\ell$ generator is employed (see Fig. 8) the same reasoning applies, but with one important difference: the discrepancy between Py8.2 and Py6.4 is not negligible and leads to a large $m_{Wb_j}^{\max }$ displacement when smearing is applied, similar to what we found at the NLO+PS level.

The $m_{Wb_j}$ and $E_{b_j}$ shifts in peak positions obtained considering several values of the jet radius *R*, with and without smearing in the case of the $m_{Wb_j}$ distribution, are summarized in Figs. 9 and 10. We notice a non-negligible *R* dependence in the difference between Py6.4 and Py8.2, both in the $hvq$ and $b\bar{b}4\ell$ case. Something similar is observed for the difference between Hw7.1 and Py8.2. A large *R* dependence is also observed in the case of Hw6.5, but with an opposite slope. The largest difference with respect to our reference result is given by the Hw7.1, that represent a major cause of concern. We stress that these large differences arise in the smeared case from the mass distribution away from the peak, i.e. cannot be consider as an irreducible uncertainty on the extracted mass.

In Fig. 9, we also see a rather striking difference between Hw7.1 and Hw6.5 interfaced to the $b\bar{b}4\ell$ generator, represented by the blue and orange square dots in the figure. The two shower generators yield differences larger than 1 GeV for the largest value of *R*. Furthermore, the *R* dependence in the two cases is opposite, in spite of the fact that, in the similar plot without hadronization and MPI (see Fig. 5), the two generators yield rather consistent results.

Overall, we find that $b\bar{b}4\ell$ and $hvq$ showered with Pythia exhibit more consistency than those showered with both versions of Herwig. This is perhaps not surprising. Matrix-element corrections (MEC), that have a large impact on $hvq$ predictions (since this generator implements only LO top decay), as implemented in the context of angular ordered parton showers (i.e. in Herwig), are technically quite different from the way in which the hardest top radiation is generated in $b\bar{b}4\ell$, at variance with MEC in transverse-momentum ordered showers (i.e. in Pythia). We find that it is difficult to use this difference to dismiss the Hw7.1 result, since the MEC formalism in Herwig has formally the same accuracy as the one in Pythia.

## Leptonic observables

The last class of observables we consider are the leptonic ones. In Ref. [1] we found that these observables are only mildly affected by non-perturbative effects (i.e. the hadronization and the MPI), thus we present only the results obtained at the full level and with jet radius $R=0.5$. However, they are likely to be strongly affected by the parton shower, since the *W* boson, and thus the leptons arising from its decay, must absorb the radiation recoil to ensure four-momentum conservation.

We extract the top mass value from the following observables:

1 $$\begin{aligned}&\langle p_{\mathchoice{\displaystyle }{\scriptstyle }{\scriptscriptstyle }{\scriptscriptstyle } \mathrm T} (\ell ^+) \rangle , \quad \langle p_{\mathchoice{\displaystyle }{\scriptstyle }{\scriptscriptstyle }{\scriptscriptstyle } \mathrm T} (\ell ^+\ell ^-) \rangle ,\quad \langle m(\ell ^+\ell ^-) \rangle ,\nonumber \\&\quad \langle E(\ell ^+\ell ^-) \rangle , \quad \langle p_{\mathchoice{\displaystyle }{\scriptstyle }{\scriptscriptstyle }{\scriptscriptstyle } \mathrm T} (\ell ^+)+ p_{\mathchoice{\displaystyle }{\scriptstyle }{\scriptscriptstyle }{\scriptscriptstyle } \mathrm T} (\ell ^-)\rangle . \end{aligned}$$

The results are presented in Table 7 and their graphical display is given in Fig. 11.

**Table 7:** Extracted mass for the $b\bar{b}4\ell$ (left) and $hvq$ (right) generators matched with Py8.2, Py6.4, Hw7.1 and Hw6.5 using the average value of the five leptonic observables. The average result is also shown

Observable	$m_{t}$ extracted with $b\bar{b}4\ell$ (GeV)				$m_{t}$ extracted with $hvq$ (GeV)			
	Py8.2	Py6.4	Hw7.1	Hw6.5	Py8.2	Py6.4	Hw7.1	Hw6.5
$\langle p_{\mathchoice{\displaystyle }{\scriptstyle }{\scriptscriptstyle }{\scriptscriptstyle } \mathrm T}(\ell ^+)\rangle$	$172.500_{- 0.825}^{+ 0.845}$	$173.649_{- 0.837}^{+ 0.867}$	$175.340_{- 0.841}^{+ 0.884}$	$176.932_{- 0.836}^{+ 0.882}$	$172.060_{- 0.811}^{+ 0.822}$	$172.847_{- 0.816}^{+ 0.850}$	$173.817_{- 0.803}^{+ 0.843}$	$175.906_{- 0.822}^{+ 0.874}$
$\langle p_{\mathchoice{\displaystyle }{\scriptstyle }{\scriptscriptstyle }{\scriptscriptstyle } \mathrm T}(\ell ^+\ell ^-)\rangle$	$172.500_{- 2.515}^{+ 1.601}$	$174.013_{- 2.282}^{+ 1.466}$	$176.328_{- 2.088}^{+ 1.353}$	$176.326_{- 2.147}^{+ 1.386}$	$174.451_{- 1.967}^{+ 1.334}$	$175.305_{- 1.809}^{+ 1.236}$	$176.675_{- 1.663}^{+ 1.141}$	$176.888_{- 1.611}^{+ 1.110}$
$\langle m(\ell ^+\ell ^-)\rangle$	$172.500_{- 1.419}^{+ 1.605}$	$173.523_{- 1.404}^{+ 1.543}$	$173.068_{- 1.363}^{+ 1.459}$	$179.337_{- 1.397}^{+ 1.546}$	$170.945_{- 1.420}^{+ 1.450}$	$171.472_{- 1.423}^{+ 1.446}$	$171.379_{- 1.412}^{+ 1.429}$	$176.330_{- 1.386}^{+ 1.458}$
$\langle E(\ell ^+\ell ^-)\rangle$	$172.500_{- 2.037}^{+ 2.061}$	$173.826_{- 2.042}^{+ 2.066}$	$174.771_{- 2.014}^{+ 2.038}$	$178.204_{- 2.017}^{+ 2.040}$	$172.490_{- 2.086}^{+ 2.076}$	$173.185_{- 2.083}^{+ 2.074}$	$173.720_{- 2.052}^{+ 2.045}$	$176.454_{- 2.039}^{+ 2.034}$
$\langle p_{\mathchoice{\displaystyle }{\scriptstyle }{\scriptscriptstyle }{\scriptscriptstyle } \mathrm T}(\ell ^+)+p_{\mathchoice{\displaystyle }{\scriptstyle }{\scriptscriptstyle }{\scriptscriptstyle } \mathrm T}(\ell ^-)\rangle$	$172.500_{- 0.827}^{+ 0.852}$	$173.680_{- 0.835}^{+ 0.867}$	$175.178_{- 0.843}^{+ 0.890}$	$177.362_{- 0.829}^{+ 0.871}$	$172.233_{- 0.802}^{+ 0.821}$	$172.940_{- 0.811}^{+ 0.846}$	$173.851_{- 0.805}^{+ 0.847}$	$175.794_{- 0.820}^{+ 0.872}$
Average	$$ {172.500_{- 0.772}^{+ 0.794} }$$	$$ {173.673_{- 0.781}^{+ 0.810} }$$	$$ {175.354_{- 0.787}^{+ 0.821} }$$	$$ {177.031_{- 0.778}^{+ 0.816} }$$	$$ {172.247_{- 0.753}^{+ 0.766} }$$	$$ {173.069_{- 0.760}^{+ 0.781} }$$	$$ {174.129_{- 0.752}^{+ 0.766} }$$	$$ {175.979_{- 0.769}^{+ 0.778} }$$

**Fig. 11:**
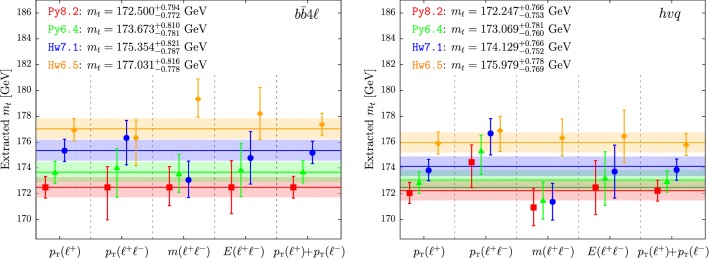
Extracted mass for the $b\bar{b}4\ell$ (left) and $hvq$ (right) generators matched with Py8.2 (red), Py6.4 (green), Hw7.1 (blue) and Hw6.5 (orange) using the average value of the five leptonic observables. The horizontal band represents the weighted average of the results, and the black horizontal line corresponds to $m_{t}=172.5$ GeV, which is the top mass value used in the $b\bar{b}4\ell$+Py8.2 reference sample

As before, our pseudo data sample was generated with $b\bar{b}4\ell$+Py8.2, and we used all combinations of NLO+PS generators and shower programs to extract a corresponding top-mass value. We remark that the mass extraction performed with the $b\bar{b}4\ell$+Py8.2 generators has been carried out using the same sample generated as pseudo data, so that the central value of the extracted mass is identical to the input mass in this case.

We have included the standard theoretical uncertainties as described in Ref. [1], and averaged the results obtained for the different leptonic observables also considering the statistical correlation among them, as suggested in Ref. [13].

The Py6.4 predictions always give $m_t$ values roughly 1 GeV larger (1.2 GeV for $b\bar{b}4\ell$ and 0.8 GeV for $hvq$) than the corresponding Py8.2 ones. This variation is of the same order of the extracted total uncertainty on $m_t$.

The average reconstructed top mass with Hw6.5 is nearly 2 GeV larger than Hw7.1 (1.8 GeV for $b\bar{b}4\ell$ and 2 GeV for $hvq$).

## Conclusions

In this work we have extended the study performed in Ref. [1] by also considering the Py6.4 and Hw6.5 generators.

**Fig. 12:**
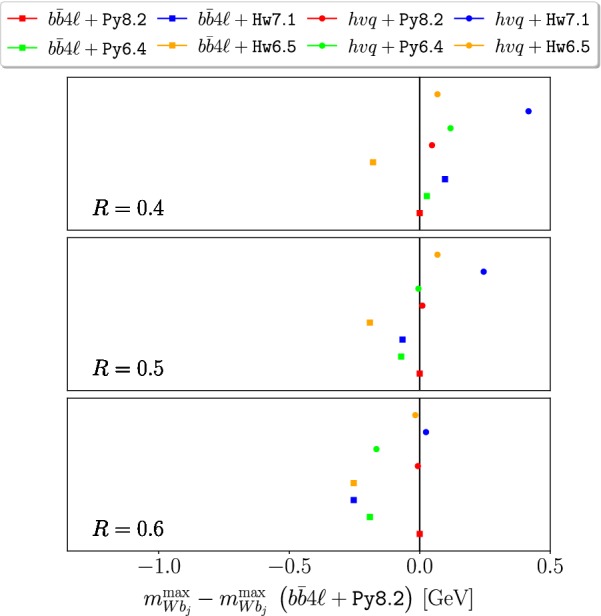
Results for the difference of the $m_{Wb_j}^{\max }$ at the Monte Carlo truth level (i.e. with no smearing) with respect to our reference generator (i.e. $b\bar{b}4\ell$+Py8.2) using $hvq$ or $b\bar{b}4\ell$, showered by Pythia and Herwig, for different values of jet radius *R*. Hadronization and MPI effects are included. The numerical values are reported in Table 9

We find that, at the NLO+PS level, the Py6.4 and Py8.2 generators (both based upon a $p_{\mathchoice{\displaystyle }{\scriptstyle }{\scriptscriptstyle }{\scriptscriptstyle } \mathrm T}$-ordered shower) are quite consistent among each other, and the same holds for Hw6.5 and Hw7.1 (both based upon an angular-ordered shower). When non-perturbative effects are included, we find larger differences between the old and the new Herwig versions of the PS programs, that yields a better agreement of the old Herwig version with respect to both Pythia versions (see Fig. 9).

If we compare predictions for the leptonic observables, we see that the old Herwig version is further away from our reference result then the new version.

Overall, the inclusion of the older versions of the shower generators supports what was found in Ref. [1], i.e. an indication of a large sensitivity to the shower generator in the extraction of the top mass. The fact remains that Herwig7 yields the most disturbing difference with respect to the other generators for what concerns the most important observable that we have considered, i.e. $m_{Wb_j}^{\max }$. On the other hand, we believe that the Herwig6.5 result, that is more in line with the Pythia ones, cannot be used to dismiss the Herwig7 one. In fact, it supports Herwig7 when only shower effects are considered, and only the inclusion of hadronization and MPI effects, thanks to an accidental cancellation, brings the final result in better agreement with the Pythia ones.

Since we have now compared four different shower and hadronization models, it is worth asking what kind of estimate of irreducible non-perturbative effects, potentially due to the different implementation of the shower cut-off and the matching hadronization model. We thus consider the spread of the $m_{Wb_j}^{\max }$ values obtained with all generators as a crude estimate of non-perturbative effects. Looking at Fig. 12, we see that the unsmeared results from the $b\bar{b}4\ell$ generators, taking $R=0.5$ to avoid too large hadronization effects (for small *R*) and too large MPI contamination (for large *R*), covers a range of roughly 200 MeV when switching among our four shower generators. If we take this range as an estimate of non-perturbative and subleading shower effects, we can conclude that, after all, these effects are well below presently quoted errors for direct measurements from the experimental collaborations.

**Table 8:** Results for $m_{Wb_j}^{\max }$ and $E_{b_j}^{\max }$ at the NLO+PS level, showered by Pythia and Herwig, without hadronization or MPI effects, for different values of jet radius *R*

Obs	Gen	Shower	$R=0.4$	$R=0.5$	$R=0.6$
$m_{Wb_j}^{\max }$ (GeV)	$b\bar{b}4\ell$	Py8.2	$172.509\pm 0.002$	$172.522\pm 0.002$	$172.538\pm 0.002$
		Py6.4	$172.487\pm 0.002$	$172.512\pm 0.002$	$172.538\pm 0.002$
		Hw7.1	$172.509\pm 0.002$	$172.512\pm 0.002$	$172.517\pm 0.002$
		Hw6.5	$172.503\pm 0.002$	$172.510\pm 0.002$	$172.513\pm 0.002$
	$hvq$	Py8.2	$172.485\pm 0.001$	$172.498\pm 0.001$	$172.513\pm 0.001$
		Py6.4	$172.475\pm 0.001$	$172.499\pm 0.001$	$172.527\pm 0.001$
		Hw7.1	$172.497\pm 0.001$	$172.498\pm 0.001$	$172.499\pm 0.001$
		Hw6.5	$172.495\pm 0.001$	$172.497\pm 0.001$	$172.500\pm 0.001$
$m_{Wb_j}^{\max }$ (GeV) smearing	$b\bar{b}4\ell$	Py8.2	$170.569\pm 0.002$	$171.403\pm 0.002$	$172.117\pm 0.002$
		Py6.4	$170.274\pm 0.002$	$171.118\pm 0.002$	$171.859\pm 0.002$
		Hw7.1	$169.699\pm 0.002$	$170.419\pm 0.002$	$171.108\pm 0.002$
		Hw6.5	$169.633\pm 0.002$	$170.454\pm 0.002$	$171.225\pm 0.002$
	$hvq$	Py8.2	$170.518\pm 0.001$	$171.315\pm 0.001$	$171.996\pm 0.001$
		Py6.4	$170.594\pm 0.001$	$171.384\pm 0.001$	$172.064\pm 0.001$
		Hw7.1	$170.464\pm 0.001$	$171.202\pm 0.001$	$171.867\pm 0.001$
		Hw6.5	$170.560\pm 0.001$	$171.283\pm 0.001$	$171.953\pm 0.001$
$E_{b_j}^{\max }$ (GeV)	$b\bar{b}4\ell$	Py8.2	$67.145\pm 0.086$	$69.614\pm 0.082$	$71.747\pm 0.080$
		Py6.4	$66.722\pm 0.089$	$69.115\pm 0.084$	$71.235\pm 0.083$
		Hw7.1	$65.847\pm 0.084$	$67.948\pm 0.083$	$69.945\pm 0.082$
		Hw6.5	$65.616\pm 0.084$	$67.703\pm 0.083$	$69.928\pm 0.083$
	$hvq$	Py8.2	$66.791\pm 0.068$	$69.357\pm 0.063$	$71.598\pm 0.061$
		Py6.4	$66.768\pm 0.067$	$69.257\pm 0.065$	$71.465\pm 0.062$
		Hw7.1	$66.276\pm 0.065$	$68.650\pm 0.063$	$70.819\pm 0.061$
		Hw6.5	$66.699\pm 0.061$	$68.923\pm 0.060$	$71.000\pm 0.057$

## Data Availability

This manuscript has no associated data or the data will not be deposited. [Authors’ comment: Our manuscript has no associated data. There are no external data associated with the manuscript.]

## Appendix A: Numerical results

In this section we give the numerical results for the hadronic observables $m_{Wb_j}^{\max }$ and $E_{b_j}^{\max }$ for both the $hvq$ and the $b\bar{b}4\ell$ generators, showered with Py8.2, Py6.4, Hw7.1 and Hw6.5. In Table 8 the results obtained without the inclusion of the hadronization and MPI effects are listed. The graphical representation of these data is given in Figs. 5 and 6.

The results obtained including the non-perturbative physics effects are instead reported in Table 9 and displayed in Figs. 9 and 10.

**Table 9:** $m_{Wb_j}^{\max }$ and $E_{b_j}^{\max }$ results at the full level, i.e. with the inclusion of the MPI and of the hadronization

Obs	gen	shower	$R=0.4$	$R=0.5$	$R=0.6$
$m_{Wb_j}^{\max }$ (GeV)	$b\bar{b}4\ell$	Py8.2	$172.156\pm 0.004$	$172.793\pm 0.004$	$173.436\pm 0.005$
		Py6.4	$172.183\pm 0.004$	$172.722\pm 0.004$	$173.245\pm 0.005$
		Hw7.1	$172.253\pm 0.005$	$172.727\pm 0.005$	$173.183\pm 0.006$
		Hw6.5	$171.977\pm 0.005$	$172.602\pm 0.006$	$173.183\pm 0.007$
	$hvq$	Py8.2	$172.203\pm 0.003$	$172.803\pm 0.003$	$173.429\pm 0.004$
		Py6.4	$172.274\pm 0.003$	$172.788\pm 0.003$	$173.270\pm 0.004$
		Hw7.1	$172.573\pm 0.004$	$173.038\pm 0.004$	$173.460\pm 0.004$
		Hw6.5	$172.224\pm 0.004$	$172.861\pm 0.004$	$173.419\pm 0.005$
$m_{Wb_j}^{\max }$ (GeV) smearing	$b\bar{b}4\ell$	Py8.2	$171.018\pm 0.002$	$172.717\pm 0.002$	$174.378\pm 0.002$
		Py6.4	$170.718\pm 0.002$	$172.270\pm 0.002$	$173.775\pm 0.002$
		Hw7.1	$170.188\pm 0.002$	$171.626\pm 0.002$	$173.111\pm 0.002$
		Hw6.5	$170.549\pm 0.002$	$172.409\pm 0.002$	$174.289\pm 0.002$
	$hvq$	Py8.2	$170.905\pm 0.001$	$172.570\pm 0.001$	$174.203\pm 0.001$
		Py6.4	$170.948\pm 0.001$	$172.459\pm 0.001$	$173.918\pm 0.001$
		Hw7.1	$170.833\pm 0.001$	$172.319\pm 0.001$	$173.814\pm 0.001$
		Hw6.5	$171.124\pm 0.001$	$172.991\pm 0.001$	$174.851\pm 0.001$
$E_{b_j}^{\max }$ (GeV)	$b\bar{b}4\ell$	Py8.2	$67.792\pm 0.089$	$71.200\pm 0.081$	$74.454\pm 0.076$
		Py6.4	$67.326\pm 0.087$	$70.415\pm 0.084$	$73.495\pm 0.081$
		Hw7.1	$66.162\pm 0.083$	$69.050\pm 0.081$	$72.098\pm 0.083$
		Hw6.5	$67.162\pm 0.084$	$70.436\pm 0.081$	$73.957\pm 0.081$
	$hvq$	Py8.2	$67.230\pm 0.066$	$70.744\pm 0.064$	$74.131\pm 0.060$
		Py6.4	$67.361\pm 0.066$	$70.558\pm 0.062$	$73.658\pm 0.061$
		Hw7.1	$66.468\pm 0.065$	$69.716\pm 0.062$	$72.943\pm 0.062$
		Hw6.5	$67.790\pm 0.060$	$71.113\pm 0.058$	$74.622\pm 0.057$

## Footnotes

1 We achieve this by setting the Py6.4 parameter PARP(71)=4 rather than the default Perugia value PARP(71)=2.

2 See eqs. (7.2) and (7.4) of Ref. [1].

3 Further studies suggest that this bump is a symptom of a minor shower cut-off mismatch between Hw7.1 and $b\bar{b}4\ell$.

4 In Hw6.5 the variable *z* is interpreted as the energy fraction of the emitter after the emission, while in Hw7.1 it represents the light-cone momentum fraction. In both, the ordering variable in the collinear limit becomes $\sim E \theta$, *E* being the energy of the emitting parton and $\theta$ the angle between the two radiated partons. See [10] for further details.

5 We stress that, among other improvements over Hw6.5, Hw7.1 implements a model for the treatment of colour reconnection.
